# First identification of *Cytauxzoon manul* in Eurasian lynx (*Lynx lynx*) in northwestern China

**DOI:** 10.1186/s13071-024-06326-1

**Published:** 2024-06-06

**Authors:** Nannan Cui, Lixin Su, Ziqi Wang, Sándor Hornok, Lijuan Tang, Meihua Yang, Yujiang Zhang, Guoyu Zhao, Yuanzhi Wang

**Affiliations:** 1 Key Laboratory for Prevention and Control of Emerging Infectious Diseases and Public Health Security of the XPCC, School of Medicine, Shihezi University, Shihezi, 832002 Xinjiang Uygur Autonomous Region People’s Republic of China; 2 Department of Parasitology and Zoology, University of Veterinary Medicine, Budapest, Hungary; 3 HUN-REN-UVMB Climate Change: New Blood-Sucking Parasites and Vector-Borne Pathogens Research Group, Budapest, Hungary; 4 Bayingolin Vocational and Technical College, Korla, 841000 Xinjiang Uygur Autonomous Region People’s Republic of China; 5 Department of Forest, College of Agriculture, Shihezi University, Shihezi, 832002 Xinjiang Uygur Autonomous Region People’s Republic of China; 6 Xinjiang Key Laboratory of Vector-Borne Infectious Diseases, Xinjiang Center for Disease Control and Prevention, Urumqi, 830002 People’s Republic of China

**Keywords:** Eurasian lynx, *Cytauxzoon manul*, *Hepatozoon felis*, Northwestern China

## Abstract

**Background:**

Multiple species of the genera *Cytauxzoon* and *Hepatozoon* can infect wild felines, but the diversity of these and other apicomplexan parasites in Eurasian lynx is scarcely known. The aim of this study was to detect *Cytauxzoon* and *Hepatozoon* species with molecular methods in Eurasian lynxes and their ticks in northwestern China.

**Methods:**

DNA was extracted from the heart, liver, spleen, lung, and kidney samples of three Eurasian lynxes as well as from their five ixodid ticks. These DNA samples were screened with polymerase chain reactions (PCRs) for *Cytauxzoon* with the partial cytochrome b gene (*CytB*), cytochrome c oxidase subunit I gene (*COI*), and small subunit ribosomal RNA gene (*18S rRNA*), and *Hepatozoon* with three different fragments of small subunit ribosomal RNA gene (*18S rRNA*). PCR products were sequenced, aligned, and phylogenetically analyzed.

**Results:**

One adult female of Eurasian lynx (#1, adult female) was co-infected with *Cytauxzoon manul* and *Hepatozoon felis* genotype I, while an adult male lynx (#2) was infected with *C. manul*. Interestingly, *H. felis* genotype I was both detected in a male cub (#3) and two out of five infesting *Hyalomma asiaticum* ticks.

**Conclusions:**

For the first time, *Cytauxzoon manul* is reported here from Eurasian lynx. In addition, *H. felis* has not been known to occur in this host species in China and Central Asia. Thus, the findings of this study extend our knowledge on the geographical distribution and host range of these haemoprotozoan parasites. Moreover, this is also the first evidence of *C. manul* and *H. felis* co-infection in Eurasian lynx.

**Graphical Abstract:**

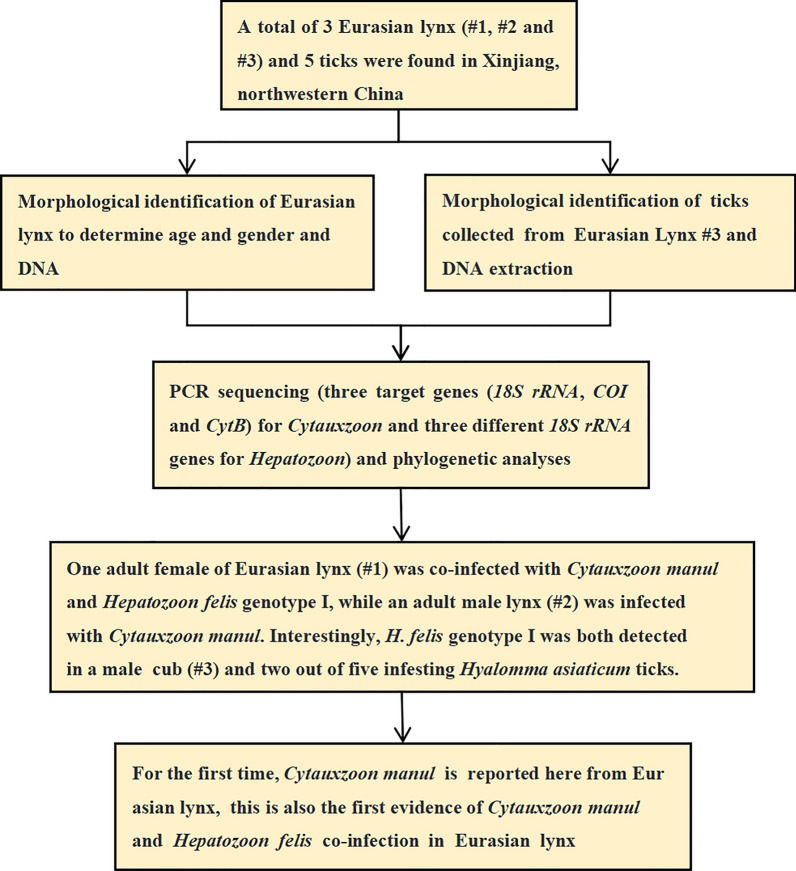

**Supplementary Information:**

The online version contains supplementary material available at 10.1186/s13071-024-06326-1.

## Background

Vector-borne pathogens are the causative agents of several emerging or re-emerging infectious diseases among felines [1, 2]. Nowadays, their epidemiological patterns undergo changes, their geographical ranges tend to expand, and their global incidence rates also increase owing to climatic change and various environmental, demographic and human-related factors [3].

Cytauxzoonosis is an emerging tick-borne disease of domestic cats and wild felids, caused by members of the genus *Cytauxzoon* (Apicomplexa: Aconoidasida: Piroplasmida: Theileriidae) [4, 5]. The clinic-pathologically most important species, *Cytauxzoon felis* was described from domestic cat [5], and was thought to be endemic only to North America, specifically in the southern, southeastern, and mid-Atlantic regions of the USA [3, 6]. In North America, the bobcat (*Lynx rufus*) is the most common natural host for *C. felis*. Bobcats usually experience a brief and mild illness upon infection, followed by a full recovery [7–9]. More recently, *Cytauxzoon* spp., infecting Eurasian lynx and wild cats in Eurasia, were reported [10].

Hepatozoonosis is a parasitic disease caused by members of the genus *Hepatozoon* (Apicomplexa: Conoidasida: Coccidia: Eucoccidiorida: Adeleorina: Hepatozoidae). These protozoa infect various mammals, birds, reptiles, and amphibians [11]. Among felids, *Hepatozoon felis* and other species can cause anorexia, pale mucous membranes, weight loss, pain, diarrhea, vomiting, gait abnormalities, fever, polyuria, polydipsia, and even death in severe cases [12–14]. The life cycle of *Hepatozoon* spp. involves a vertebrate host, which gets infected by ingesting arthropod vectors (e.g., ticks) [13, 15, 16]. The infection can also occur by predation and transplacental transmission [17–19].

In this study, *Cytauxzoon* and *Hepatozoon* spp. were molecularly screened from Eurasian lynx (*Lynx lynx*) and their ixodid ticks in northwestern China.

## Methods

### Sample collection

Three Eurasian lynxes were investigated in this study. According to their anatomy characteristics, body weight, and tooth wear, the sex and age of three lynxes were evaluated [20]. Two of them, an adult female (#1, 4–5 years old) and an adult male (#2, 3–4 years old), were found dead due to natural causes during our field investigation at the China–Kazakhstan border at the West Junggar Mountain in 2018 and 2019, respectively. The third one, a road-killed male cub (#3, 4–6 months old), was also collected in this region in 2019 as already reported in Liu et al. [21]. Five ticks were collected from the male cub, the latter, and molecularly identified as *Hyalomma asiaticum* [22].

### DNA extraction

Genomic DNA was individually extracted from heart, liver, spleen, lung, and kidney samples of three lynxes. The sampled ticks were carefully surface-sterilized, and prior to processing, the exterior of all ticks was disinfected using 3% sodium hypochlorite for 1 min, 70% ethanol for 1 min, and phosphate-buffered saline (PBS) for 1 min. DNA was extracted from whole ticks using TIANamp Genomic DNA Kit (TIANGEN, Beijing, China), with an overnight following the manufacturer’s instructions. DNA extracts were eluted in 60 μL of Tris–EDTA buffer and stored at −80 °C under sterile conditions to prevent contamination until polymerase chain reaction (PCR) analysis.

### Polymerase chain reaction amplification

DNA extracts (all from lynxes and five from ticks) were individually screened for the presence of *Cytauxzoon* and *Hepatozoon* spp. with PCR and sequencing. For genotyping *Hepatozoon* spp., 325-, 620-, and 1700-bp-long fragments of the small subunit 18S ribosomal RNA gene (*18S rRNA*) were chosen [23]. PCR was also performed using primer set of *Cytauxzoon*, targeting the partial *18S rRNA* gene fragment (900bp), 1150 bp fragment of the *CytB* gene, and 1320 bp fragment of the *COI* gene [24]. The primers and PCR cycling conditions are shown in Additional file 1. A negative control (distilled water) was included in each run to validate primer-specific amplification. The PCR products were subjected to electrophoresis in 1.5% agarose gel and visualized under ultraviolet (UV) light by staining the gel with Goldview (Biotopped, Beijing, China). All PCR products were purified using the TIANgel Midi Purification Kit (TIANGEN, Beijing, China) and sequenced by Sangon Biotech Co., Ltd. (Shanghai, China) using the same primers.

### Sequencing and data analyses

Sequencing data were subjected to Basic Local Alignment Search Tool (BLAST) searches (http://www.ncbi.nlm.nih.gov/blast/) and then aligned and analyzed with reference sequences downloaded from GenBank. Phylogenetic trees were constructed on the basis of the sequence distance method using the maximum likelihood algorithms implemented in the Molecular Evolutionary Genetics Analysis (MEGA) 7.0 software [25]. All sequences from this study were deposited in the GenBank (http://www.ncbi.nlm.nih.gov) database (*C*. *manul 18S rRNA*: PP033938;* C*. *manul CytB*: PP442054; *C*. *manul COI*: PP503316; *H*. *felis 18S rRNA*: PP033238, PP528680-PP528683, OR497518, and OR497519).

## Results

**Fig. 1:**
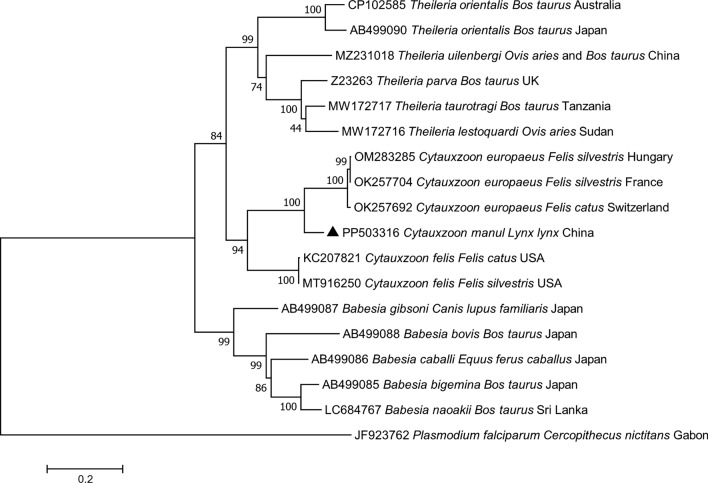
Phylogenetic tree based on *COI* gene sequences of *Cytauxzoon manul* (filled triangle) from Eurasian lynx, constructed with the maximum likelihood method and using the General Time Reversible model with discrete Gamma distributed with invariant sites (bootstrap replicates: 1000). The GenBank accession number, strain name, host, and area of origin were listed. *Plasmodium falciparum* was used as an outgroup

**Fig. 2:**
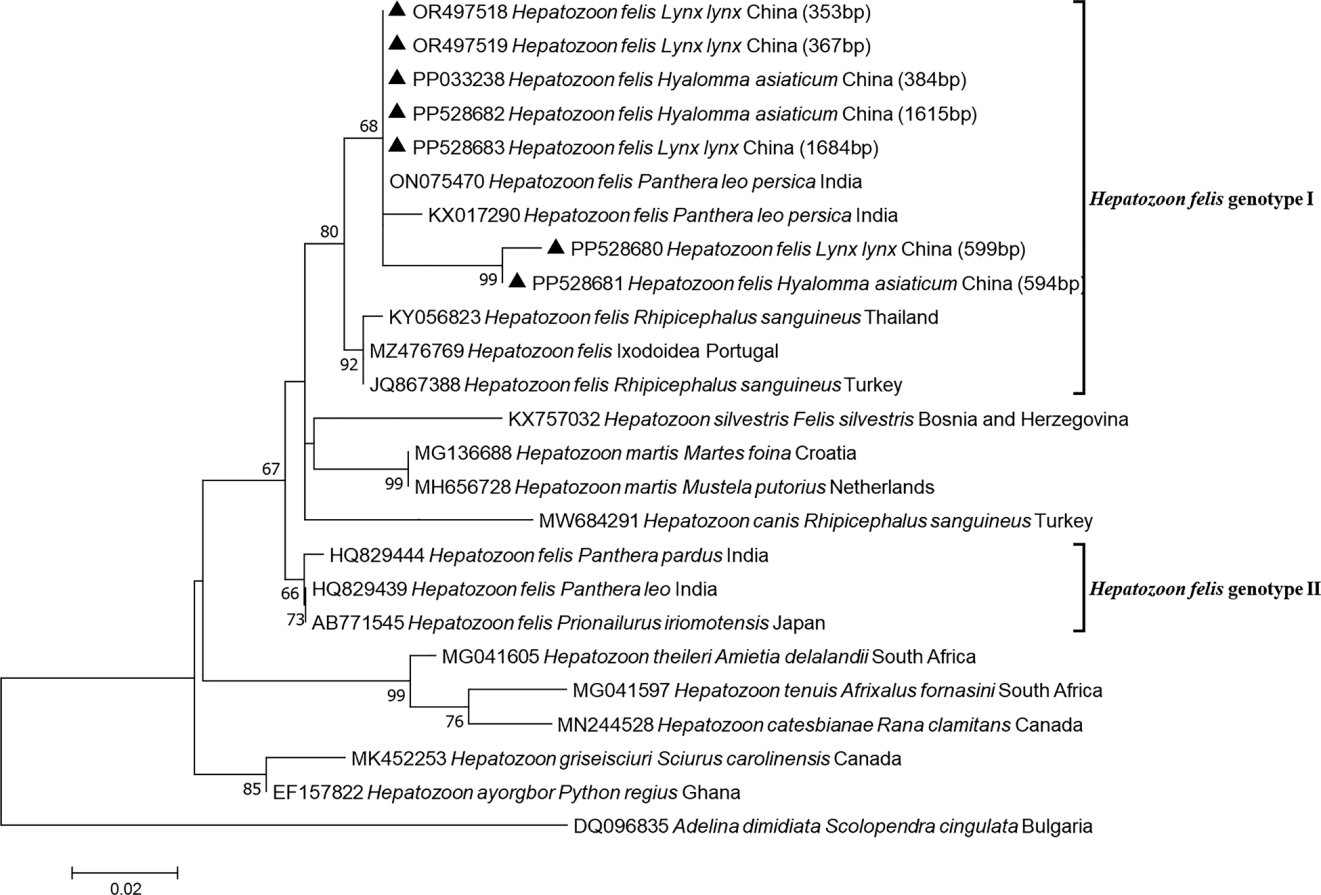
Phylogenetic tree based on *18S rRNA* gene sequences of *Hepatozoon felis* (filled triangle) from Eurasian lynx, constructed with the maximum likelihood method and using the Tamura 3-parameter substitution model with discrete gamma distribution (bootstrap replicates: 1000). The GenBank accession number, strain name, host, and area of origin were listed. *Adelina dimidiata* was used as an outgroup

The results of molecular analysis and sequencing indicated that (i) Eurasian lynx #1 was found to be coinfected with *Cytauxzoon manul* and *Hepatozoon felis*; (ii) Eurasian lynxes #2 and #3 were infected with a *H. felis* genotype I that was significantly the same from that in *Hyalomma asiaticum* infesting Eurasian lynx cub #3. Phylogenetic trees and BLAST analyses showed that *C*. *manul 18S rRNA* gene sequences from this study were grouped with those from Pallas’s cat (*Otocolobus manul*) in Mongolia (shown in Additional file 2: Fig. S1), and shared 99.89% (871/872) of identities with sequences available in GenBank (AY485690 and AY485691). To assess the genetic variability of *Cytauxzoon* spp., sequence analyses of mitochondrial *COI* gene were performed. The results of our phylogenetic analyses indicated a sister group relationship among *C. manul* and *Cytauxzoon* spp. from felines (Fig. 1), which was similar to the result based on *CytB* gene (shown in Additional file 3: Fig. S2). The phylogenetic tree of *Hepatozoon* spp. based on the partial *18S rRNA* gene fragment showed that this genotype was clustered into the clade of “genogroup I”, separately from other isolates of which corresponding sequences are available in GenBank (Fig. 2), and shared 99.94% (1684/1685 bp) of identities with those from Asiatic lion in India (ON075470).

## Discussion

This study provides the first evidence for the occurrence of *C. manul* and *H. felis* in Eurasian lynx, and this is the first time that infection with *H. felis* genotype I has been reported in Eurasian lynx and *Hyalomma asiaticum* ticks in China. To the best of our knowledge, this is also the first report of *C. manul* and *H. felis* co-infection in Felid.

Cytauxzoonosis, caused by *C. felis*, *C. manul*, and three recently described new *Cytauxzoon* European species, is an emerging infectious disease that affects wild felids as well as the domestic cat [24, 26, 27]. *Cytauxzoon manul* is endemic in free-ranging Pallas’s cats (*Otocolobus manul*) in Mongolia [28], and it was also reported from lions (*Panthera leo*) in Zimbabwe [29]. More recently, *Cytauxzoon* spp., different from *C. manul*, were detected in Romania in four Eurasian lynxes (*Lynx lynx*) and 12 wild cats (*Felis silvestris*) [10]. This study reports the first detection of *C. manul* in Eurasian lynx. Currently, study on *C. manul* is scarce. In the future, more research is needed to characterize the epidemiology of this species.

Three *Hepatozoon* species are known to infect felines, including *Hepatozoon felis*, *Hepatozoon canis*, and *Hepatozoon silvestris* [19, 23, 30]. *Hepatozoon felis* was previously detected in wild cat in the Republic of Cape Verde, in jaguar (*Panthera onca*) and in jaguarundi (*Puma yagouaroundi*) in Brazil, in leopard cat (*Prionailurus bengalensis*) in Korea, and in Eurasian lynx (*Lynx lynx*) in Turkey, as well as in Asiatic lion (*Panthera leopersica*), Indian tiger (*Panthera tigris tigris*), and Indian leopard (*Panthera pardus fusca*) in India [31–36]. *H*. *felis* was reported in *Haemaphysalis longicornis* (Acari: Ixodidae) ticks from free-ranging domestic sheep in Hebei Province, China [37]. In the present study, *H. felis* genotype I was detected both in a lynx cub and its infecting *Hy*. *asiaticum* ticks.

In this study, although *H. felis* was detected in ticks, it is still impossible determine whether these ticks are truly infected with *H. felis*. Given that these ticks were engorged, the detection of *H. felis* DNA in the blood meal was also possible. Therefore, the question of whether *Hy*. *asiaticum* ticks can be infected with *H. felis* still needs further research and confirmation.

Protozoan co-infections are relatively frequent in carnivores [38–43]. Although this phenomenon, as also observed in this study, is seldom reported in lynxes, *Babesia* sp. and *H*. *felis* were detected simultaneously in Eurasian lynx in Turkey [34]. Eurasian lynx is included in the Red List of Threatened Species by International Union for Conservation of Nature (IUCN) and is also listed on the second level of National Key Protected Wildlife in China [44]. To better understand the impact of these parasites on the health and conservation status of the Eurasian lynx, future studies should identify its complete pathogen profile by metagenomic next-generation sequencing.

## Conclusions

In this study, *C*. *manul* and *H*. *felis* genotype I were molecularly identified in Eurasian lynx. These two haemoprotozoan parasites caused co-infection in a lynx. *Hepatozoon felis* was detected both in a lynx cub and its *Hyalomma asiaticum* ticks. These finding extends our knowledge on the geographical distribution and host range of *C*. *manul* and *H*. *felis*.

### Supplementary Information

Additional file 1: Table S1. Characteristics of PCRs used in this study: target genes, primer sequences, and cycling conditions.

Additional file 2: Figure S1. Phylogenetic tree based on *18S rRNA* gene sequences of *Cytauxzoon manul* (▲) from Eurasian lynx, constructed with the maximum likelihood method and using the Tamura 3-parameter substitution model with discrete Gamma distributed with invariant sites (bootstrap replicates: 1000). The GenBank accession number, strain name, host, and area of origin were listed. *Plasmodium falciparum* was used as an outgroup.

Additional file 3: Figure S2. Phylogenetic tree based on *CytB* gene sequences of *Cytauxzoon manul* (▲) from Eurasian lynx, constructed with the maximum likelihood method and using the Hasegawa-Kishino-Yano model with discrete Gamma distributed with invariant sites (bootstrap replicates: 1000). The GenBank accession number, strain name, host, and area of origin were listed. *Plasmodium falciparum* was used as an outgroup.

## Data Availability

The sequences obtained and analyzed during the present study are deposited in the GenBank database under the accession numbers (*C. manul 18S rRNA*: PP033938; *C. manul CytB*: PP442054; *C. manul COI*: PP503316; *H. felis 18S rRNA*: PP033238, PP528680-PP528683, OR497518, and OR497519).

## Abbreviations

PCR: Polymerase chain reaction
DNA: Deoxyribonucleic acid
*18S rRNA*: 18S Ribosomal RNA
*COI*: Cytochrome c oxidase subunit I
*CytB*: Cytochrome B
