# Supplementary material for: First identification of Cytauxzoon manul in Eurasian lynx (Lynx lynx) in northwestern China
Source: Parasit Vectors. 2024 Jun 6;17:249. doi: 10.1186/s13071-024-06326-1 (PMC11157914; doi:10.1186/s13071-024-06326-1)
Supplement: Supplementary file 1 — Additional file 1: Table S1. Characteristics of PCRs used in this study: target genes, primer sequences, and cycling conditions. [file 13071_2024_6326_MOESM1_ESM.pdf]

Additional file 1: Table S1.

Molecular investigation of ticks and tick-borne protozoan parasites (Apicomplexa: *Cytauxzoon* and *Hepatozoon* spp.) from Eurasian lynx (*Lynx lynx*) in northwestern China: first identification of *Cytauxzoon manul* in this host species

The PCR equipment was a Mastercycler X50s, Eppendorf, Germany.

1. PCR amplification to detect *Cytauxzoon* spp. based on a 1200-bp-long part of the *18S rRNA* gene

The PCR amplifications were performed in a 25 µL reaction volume. The reaction mixture contained 0.75 µmol/L of each primer (rrf-O), 250 µM of each dNTP, and 1.0 U of Taq polymerase (TaKaRa Taq Version 2.0, Takara, Dalian, China).

Cycling conditions consisted of an initial denaturation at 94 °C for 30 sec, 35 cycles each of 94 °C for 30 sec, 60 °C for 45 sec and 72 °C for 1 min 30 sec followed by final extension at 72 °C for 5 min.

2. PCR amplification to detect *Cytauxzoon* spp. based on a 1333bp-bp-long fragment of the *CytB* gene

Cycling conditions consisted of an initial denaturation at 95 °C for 5 min; 45 cycles each of 95 °C for 20 s; 53 °C for 30 s; 68 °C for 1.5 min followed by final extension at 68 °C for 7 min;

The conditions for the second PCR consisted of an initial denaturation at 95 °C for 5 min; 45 cycles each of 95 °C for 20 s; 55 °C for 30 s; 68 °C for 1.5 min followed by final extension at 68 °C for 7 min.

3. PCR amplification to detect *Cytauxzoon* spp. based on a 1333bp-bp-long fragment of the *CytB* gene

Cycling conditions consisted of an initial denaturation 95 °C for 5 min; 40 cycles each of 94 °C for 20 s, 64 °C for 30 s,and 68 °C for 2.5

min followed by final extension at 72 °C for 7 min;

The conditions for the second PCR consisted of an initial denaturation at 95 °C for 5 min; 40 cycles each of 94 °C for 20 s, 60 °C for 30 s,and 68 °C for 2 min followed by final extension at 72 °C for 7 min.

4. PCR amplification to detect *Hepatozoon* spp. based on a 397-bp-long fragment of the *18S rRNA* gene

The PCR amplifications were performed in a 25 μL reaction volume. The reaction mixture contained 0.75 μmol/L of each primer (rrf-O), 250 μM of each dNTP, and 1.0 U of Taq polymerase (TaKaRa Taq Version 2.0, Takara, Dalian, China).

Cycling conditions consisted of an initial denaturation at 95 °C for 5 min, followed by 35 cycles at 95 °C for 30 s, 53 °C for 30s, and 72 °C for 30s, with a final extension at 72 °C for 8 min.

The conditions for the second PCR are the same.

5. PCR amplification to detect *Hepatozoon* spp. based on a 620-bp-long fragment of the *18S rRNA* gene

The PCR amplifications were performed in a 25 μL reaction volume. The reaction mixture contained 0.75 μmol/L of each primer (rrf-O), 250 μM of each dNTP, and 1.0 U of Taq polymerase (TaKaRa Taq Version 2.0, Takara, Dalian, China).

Cycling conditions consisted of an initial denaturation at 95 °C for 2 min, followed by 35 cycles at 95 °C for 1 min, 58°C for 1 min, and 72°C for 1 min, with a final extension at 72°C for 5 min.

6. PCR amplification to detect *Hepatozoon* spp. based on a 1700-bp-long fragment of the *18S rRNA* gene

The PCR amplifications were performed in a 25 μL reaction volume. The reaction mixture contained 0.75 μmol/L of each primer (rrf-O), 250 μM of each dNTP, and 1.0 U of Taq polymerase (TaKaRa Taq Version 2.0, Takara, Dalian, China).

Cycling conditions consisted of an initial denaturation at 95 °C for 2 min, followed by 35 cycles at 95°C for 30 s, 58°C for 30s, and 72°C for 1.5 min, with a final extension at 72°C for 5 min.

Characteristics of amplified fragments and corresponding primer sequences

Targeted DNA	Gene	Primer sequence (5'-3)	Fragment	Cycling conditions of PCR assays	Reference
Cytauxzoon	*18S rRNA*	Forward (CCAGCTCCAATAGCGTATATT)<br>Reverse (AGGATGAACTCGATGAATGCA)	1200 bp	initial denaturation 94 °C for 30 sec, 35 cycles each of 94 °C for 30 sec, 60 °C for 45 sec and 72 °C for 1 min 30 sec followed by final extension at 72 °C for 5 min.	[1]
	CytB	Forward 1 (CTTAACCCAACTCACGTACC)<br>Reverse 1 (GGTTAATCTTTCCTATTCCTTACG)	1434bp	95 °C for 5 min; 45× (95 °C for 20 s; 53 °C for 30 s; 68 °C for 1,5 min); 68 °C for 7 min	[2]
		Forward 2 (ACCTACTAAACCTTATTCAAGCRTT)<br>Reverse 2 (AGACTCTTAGATGYAAACTTCCC)	1333bp	95 °C for 5 min; 45× (95 °C for 20 s; 55 °C for 30 s; 68 °C for 1,5 min); 68 °C for 7 min	
	COI	Forward 1 (TGGYTKGCTTATTGGTTTGG)<br>Forward 1 (ACTTTGAACACACTGCTCG)	1966bp	95 °C for 5 min, followed by 40 cycles at 94 °C for 20 s, 64 °C for 30 s,and 68 °C for 2.5 min,with a final extension at	[2]

				72 °C for 7 min	
		Forward 2 (TGGYTKGCTTATTGGTTTGG)<br>Forward 2 (AATTCCCATCTCGCTATCACTTTC)	1656bp	95 °C for 5 min, followed by 40 cycles at 94 °C for 20 s, 60 °C for 30 s,and 68 °C for 2 min,with a final extension at 72 °C for 7 min	
	18S rRNA	Forward (ACAGCATAAGAGAGGTAGTGAC)<br>Reverse 1 (CCATGCTGCAGTATTCAAAG)<br>Reverse 1 (CCATGCTGCAGTATTCAAAG)	OUT:397 bp<br>IN: 325 bp	95 °C for 5 min, followed by 35 cycles at 95 °C for 30 s, 53 °C for 30s, and 72 °C for 30s, with a final extension at 72 °C for 8 min. The conditions for the second PCR are the same.	In this study
Hepatozoon	*18S rRNA*	Forward (GAAATAACAATCAAGGCAGTTAAAATGCT)<br>Reverse (GTGCTGAAGGAGTCGTTTATAAAGA)	620 bp	95 °C for 2 min, followed by 35 cycles at 95 °C for 1 min, 58 °C for 1 min and 72 °C for 1 min, with a final extension at 72 °C for 5 min.	[3]
	18S rRNA	Forward (GCCAGTAGTCATATGCTTGTC)<br>Reverse (GACTTCTCCTTCGTCTAAG)	1700bp	95 °C for 2 min, followed by 35 cycles at 95 °C for 30 s, 58 °C for 30 s and 72 °C for 1.5 min, with a final extension at 72 °C for 5 min	[3]

References

[1] Gallusová M, Jirsová D, Mihalca AD, Gherman CM, D'Amico G, Qablan MA, et al., *Cytauxzoon* Infections in Wild Felids from Carpathian-Danubian-Pontic Space: Further Evidence for a Different *Cytauxzoon* Species in European Felids. J Parasitol. 2016 Jun;102(3):377-80.

[2] anait LC, Mihalca AD, Modrý D, Juránková J, Ionică AM, Deak G, et al., Three new species of *Cytauxzoon* in European wild felids. Vet Parasitol. 2021 Feb; 290: 109344.

[3] Hodžić A, Alić A, Prašović S, Otranto D, Baneth G, Duscher GG. Hepatozoon silvestris sp. nov.: morphological and molecular characterization of a new species of *Hepatozoon* (Adeleorina: Hepatozoidae) from the European wild cat (*Felis silvestris silvestris*). Parasitology. 2017 Apr;144(5):650-661.
