# Supplementary figures and images for: First identification of Cytauxzoon manul in Eurasian lynx (Lynx lynx) in northwestern China
Source: Parasit Vectors. 2024 Jun 6;17:249. doi: 10.1186/s13071-024-06326-1 (PMC11157914; doi:10.1186/s13071-024-06326-1)

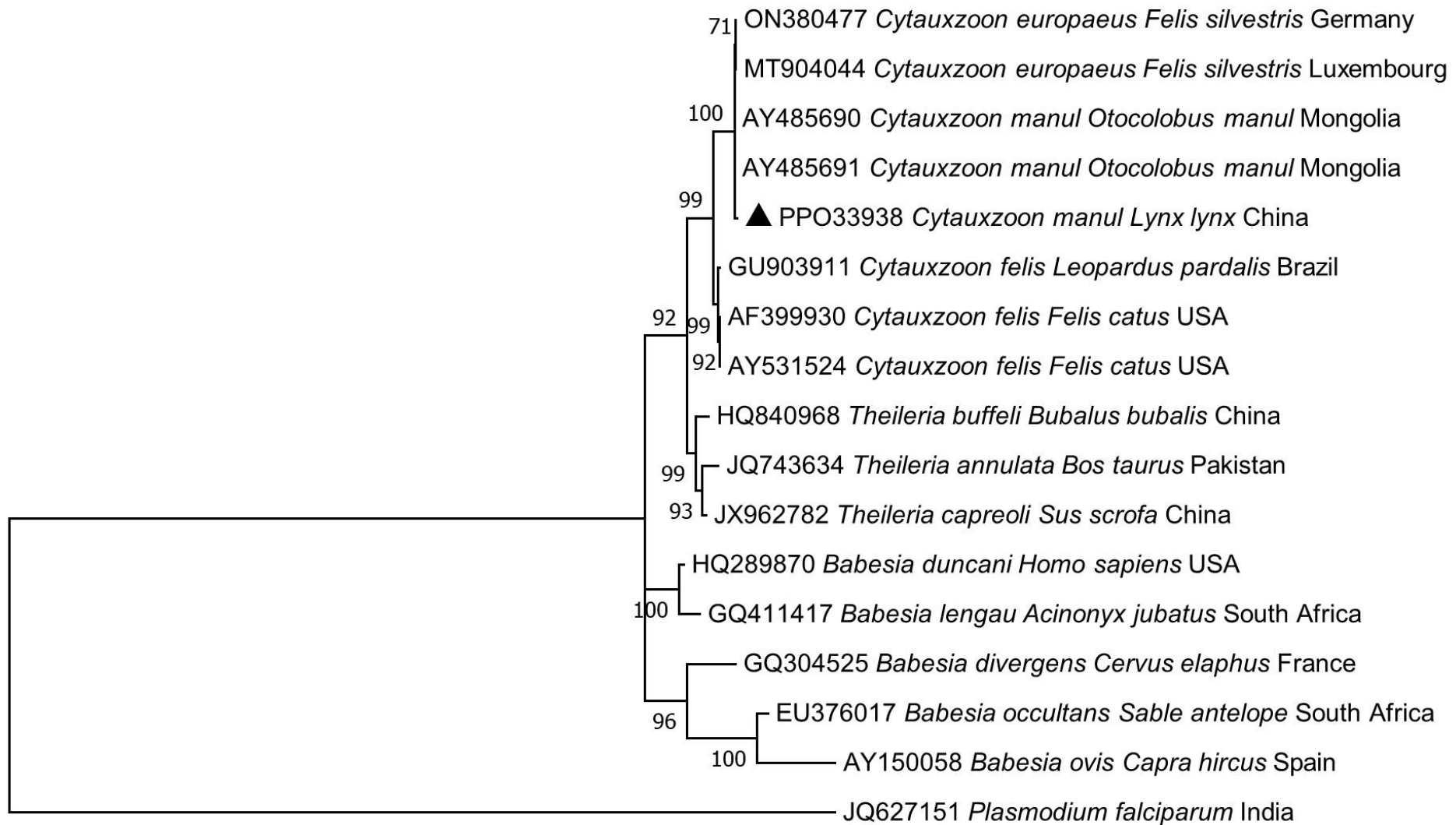

Supplement: Supplementary file 2 — Additional file 2: Figure S1. Phylogenetic tree based on 18S rRNA gene sequences of Cytauxzoon manul (▲) from Eurasian lynx, constructed with the maximum likelihood method and using the Tamura 3-parameter substitution model with discrete Gamma distributed with invariant sites (bootstrap replicates: 1000). The GenBank accession number, strain name, host, and area of origin were listed. Plasmodium falciparum was used as an outgroup. [file 13071_2024_6326_MOESM2_ESM.pdf]

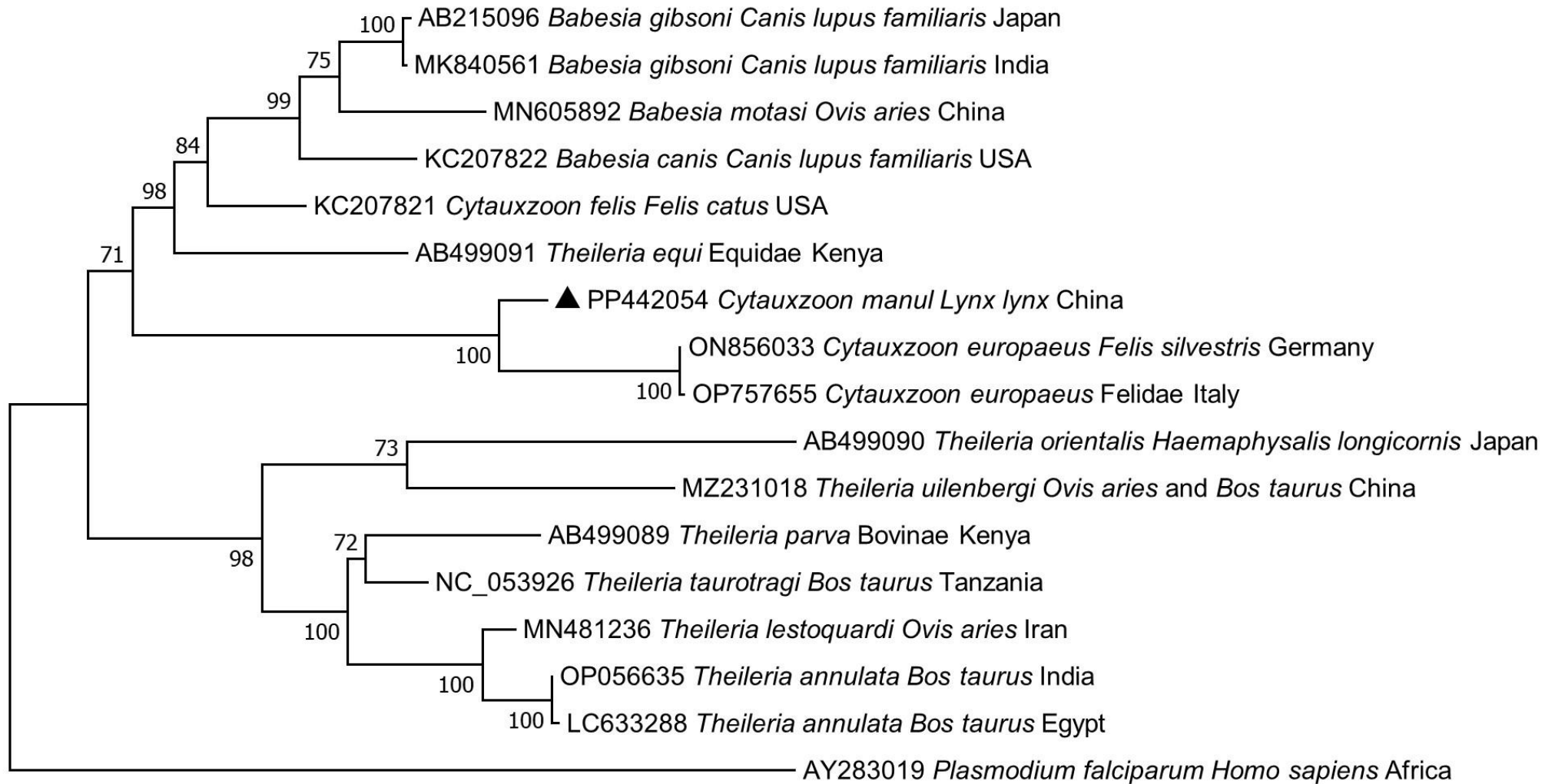

Supplement: Supplementary file 3 — Additional file 3: Figure S2. Phylogenetic tree based on CytB gene sequences of Cytauxzoon manul (▲) from Eurasian lynx, constructed with the maximum likelihood method and using the Hasegawa-Kishino-Yano model with discrete Gamma distributed with invariant sites (bootstrap replicates: 1000). The GenBank accession number, strain name, host, and area of origin were listed. Plasmodium falciparum was used as an outgroup. [file 13071_2024_6326_MOESM3_ESM.pdf]
